# Identification of genes regulated by 20-Hydroxyecdysone in *Macrobrachium nipponense* using comparative transcriptomic analysis

**DOI:** 10.1186/s12864-023-09927-9

**Published:** 2024-01-05

**Authors:** Huwei Yuan, Pengfei Cai, Wenyi Zhang, Shubo Jin, Sufei Jiang, Yiwei Xiong, Yongsheng Gong, Hui Qiao, Hongtuo Fu

**Affiliations:** 1 Wuxi Fisheries College, Nanjing Agricultural University, Wuxi, 214081 China; 2 Key Laboratory of Freshwater Fisheries and Germplasm Resources Utilization, Ministry of Agriculture and Rural Affairs, Freshwater Fisheries Research Center, Chinese Academy of Fishery Sciences, Wuxi, 214081 China

**Keywords:** 20-hydroxyecdysone, Molting, Transcriptome, *Macrobrachium nipponense*

## Abstract

**Background:**

*Macrobrachium nipponense* is a freshwater prawn of economic importance in China. Its reproductive molt is crucial for seedling rearing and directly impacts the industry’s economic efficiency. 20-hydroxyecdysone (20E) controls various physiological behaviors in crustaceans, among which is the initiation of molt. Previous studies have shown that 20E plays a vital role in regulating molt and oviposition in *M. nipponense*. However, research on the molecular mechanisms underlying the reproductive molt and role of 20E in *M. nipponense* is still limited.

**Results:**

A total of 240.24 Gb of data was obtained from 18 tissue samples by transcriptome sequencing, with > 6 Gb of clean reads per sample. The efficiency of comparison with the reference transcriptome ranged from 87.05 to 92.48%. A total of 2532 differentially expressed genes (DEGs) were identified. Eighty-seven DEGs associated with molt or 20E were screened in the transcriptomes of the different tissues sampled in both the experimental and control groups. The reliability of the RNA sequencing data was confirmed using Quantitative Real-Time PCR. The expression levels of the eight strong candidate genes showed significant variation at the different stages of molt.

**Conclusion:**

This study established the first transcriptome library for the different tissues of *M. nipponense* in response to 20E and demonstrated the dominant role of 20E in the molting process of this species. The discovery of a large number of 20E-regulated strong candidate DEGs further confirms the extensive regulatory role of 20E and provides a foundation for the deeper understanding of its molecular regulatory mechanisms.

**Supplementary Information:**

The online version contains supplementary material available at 10.1186/s12864-023-09927-9.

## Introduction

*Macrobrachium nipponense* (Arthropoda, Crustacea, Decapoda) mainly inhabits the rivers and lakes of China, Japan, southeast Asia, and eastern Russia. It is o f great nutritional and economic value due to its delicious flavor and richness in proteins and trace elements. The culture of *M. nipponense* has become one of the largest and most productive freshwater prawn enterprises in China. *M. nipponense* concentrates its molt and oviposition activity during the breeding season, in the reproductive molt. As a typical economic decapod prawn, its reproductive molt is crucial for seedling rearing and directly affects the economic efficiency of the industry, but has so far not been studied.

20-hydroxyecdysone (20E) is produced by the hydrocarbonylation of ecdysone and triggers or initiates the molting process [1]. In insects, 20E elicits not only molt, but most of the other major physiological behaviors: development, metamorphosis, and growth and reproduction, among others [2–4]. The proper functioning of monandry and oviposition in *Anopheles gambiae* is contingent upon 20E, which serves as a critical regulatory factor [5]. In *Drosophila* spp., 20E is the primary hormone controlling egg maturation and ovulation [6]. 20E also plays a critical regulatory role in molt and reproduction in crustaceans. In *Exopalaemon carinicauda*, administration of 20E significantly accelerated the molting process and increased the molting rate [7]. The molt of *Daphnia magna* is regulated by 20E titers, and molt occurs only after 20E levels have fallen to basal levels. In addition, exogenous treatment with 20E can prevent oviposition and inhibit molt in *D. magna* [8, 9]. In our previous study, knocking down the Halloween gene and the nuclear receptor genes using RNA interference (RNAi) affected the 20E content of *M. nipponense*, indirectly demonstrating that 20E regulates molt or oviposition and plays a vital role in regulating the physiology of this species [10–13]. Based on these results, we carried out transcriptomic studies to further investigate the molecular mechanisms by which 20E regulates the physiology of *M. nipponense*.

In this study, the transcriptome libraries of several different tissues of *M. nipponense* in the experimental and control groups were constructed using the Illumina sequencing platform. The experimental and control groups were treated with 20E (5 µg/g) and ethanol solution (20E 0 µg/g), respectively. The differentially expressed genes (DEGs) were screened using transcriptome comparison and annotated using various databases. We focused on screening the DEGs associated with molt or 20E in the different *M. nipponense* tissues. The accuracy of the transcriptome data was verified using Quantitative Real-Time PCR (qRT-PCR) and the expression of important candidate genes at the different stages of molt of was examined. We established the first transcriptome library of the different tissues of *M. nipponense*, from the perspective of 20E. This provided a valuable data base for future studies of the molecular mechanism of 20E in regulating the molt of *M. nipponense*, and other crustaceans.

## Materials and methods

### Experimental design

The *M. nipponense* used in the experiment were provided by the Dapu Experimental Station of the Freshwater Fisheries Research Centre, the Chinese Academy of Fisheries Sciences (Dapu, Wuxi). Before the experiments began, the prawns were acclimated in constantly circulating water (26 ± 1℃) for one week and were fed with snails twice daily (at 8:00 and 18:00). Three hundred vigorous adult female prawns (2.10 ± 0.45 g) were selected and allocated to six tanks at random. The prawns were divided into experimental and control groups. Prawns in the experimental group were injected with 5 µg/g of 20E (Sigma-Aldrich, St Louis, MO, USA) and those in the control group were injected with an equal quantity of ethanol solution (20E, 0 µg/g ), as described in our previous study [13].

### Sample collection

The prawns were fasted for 24 h before starting the injection experiment. Three hours after injection, tissues (gill, G; muscle, M; ovary, O) from the experimental and control groups were sampled from 10 prawns in each group. The tissues collected were first snap frozen using liquid nitrogen and then stored at − 80 °C in a refrigerator for subsequent RNA extraction.

### RNA extraction, cDNA library construction, and sequencing

Total RNA was extracted from the experimental and control prawn tissues using an RNAiso Plus Kit (TaKaRa, Tokyo, Japan) according to the manufacturer’s instructions. The quality of the RNA collected was assessed using three methods [14–16]: 1.2% agarose gel electrophoresis was used to detect whether the RNA was degraded or contaminated; a NanoDrop ND2000 (NanoDrop Technologies, Wilmington, DE, USA) was used to determine the purity of the RNA (OD260/280 and OD260/230); and an Agilent 2100 Bioanalyzer was used to check the integrity of the RNA (Agilent technologies, Santa Clara, CA, USA). As detailed in our previous paper, libraries were prepared using 3 µg of mRNA (concentration > 300 ng/µL) per sample [17]. Briefly, cDNA was synthesized using mRNA as a template and subsequently purified using AMPure XP Beads (Beckman Coulter, Beverly, MA, USA). The purified cDNA was then end-repaired and poly(A) tails were added and sequenced, followed by fragment size selection using AMPure XP beads. Finally, the cDNA library was enriched using PCR. Libraries were tested for quality and qualified samples were sequenced using an Illumina HiSeq 2500 high-throughput sequencing platform (Illumina Inc., San Diego, CA, USA), with a sequencing read length of PE150.

### Sequencing data quality control and annotation

The raw sequencing data were filtered to obtain high quality clean data. Mapped data were obtained by sequence alignment of the clean data with the *M. nipponense* reference genome (https://ftp.cngb.org/pub/CNSA/data2/CNP0001186/CNS0254395/CNA0014632/) using HISAT2 [18]. The number of Fragments per kilobase of transcript per million fragments mapped (FPKM) was used as a measure of transcript or gene expression level [19]. Differential screening was performed using the DESeq2 software, with Fold Change ≥ 2 and FDR < 0.05 as the screening criteria [20]. The DEGs were annotated in the Nr, Gene Ontology (GO), Kyoto Encyclopedia of Genes and Genomes (KEGG), and Clusters of Orthologous Groups (COG) databases [21].

### qRT-PCR validation

Twelve genes were selected from the candidate DEGs, and qRT-PCR primers were designed online using Primer-BLAST (https://www.ncbi.nlm.nih.gov/tools/primer-blast/) based on the DEG sequences. The target fragments were verified using gel electrophoresis. The reaction system and procedure for the qRT-PCR followed our previous study [10]. The expression levels of the DEGs were analyzed using the 2^−ΔΔCT^ method using eukaryotic translation initiation factor 5 A as an internal reference gene [22, 23]. The list of all primers used in the qRT-PCR are shown in Table S1.

### Data analysis

The experimental data were assessed for uniformity of variance and subsequently analyzed using the SPSS 20.0 software (IBM, New York, NY, USA). The Tukey method was employed in a one-way ANOVA to compare differences among multiple groups, while an independent-samples t-test was used to analyze comparisons between two groups of data. The results were expressed as the mean ± standard deviation (SD).

## Results

### Sequencing data and comparative efficiency

A total of 240.24 Gb of data was obtained from 30 tissue samples by transcriptome sequencing, with > 6 Gb of clean reads per sample. A total of 800,987,724 pair-end reads, 161,531 transcripts, and 104,253 unigenes were obtained. The Q30 of each sample data set was > 90% and the efficiency of comparison with the reference transcriptome ranged from 87.05 − 92.48% (Table S2).

### Identification and annotation of DEGs

**Fig. 1:**
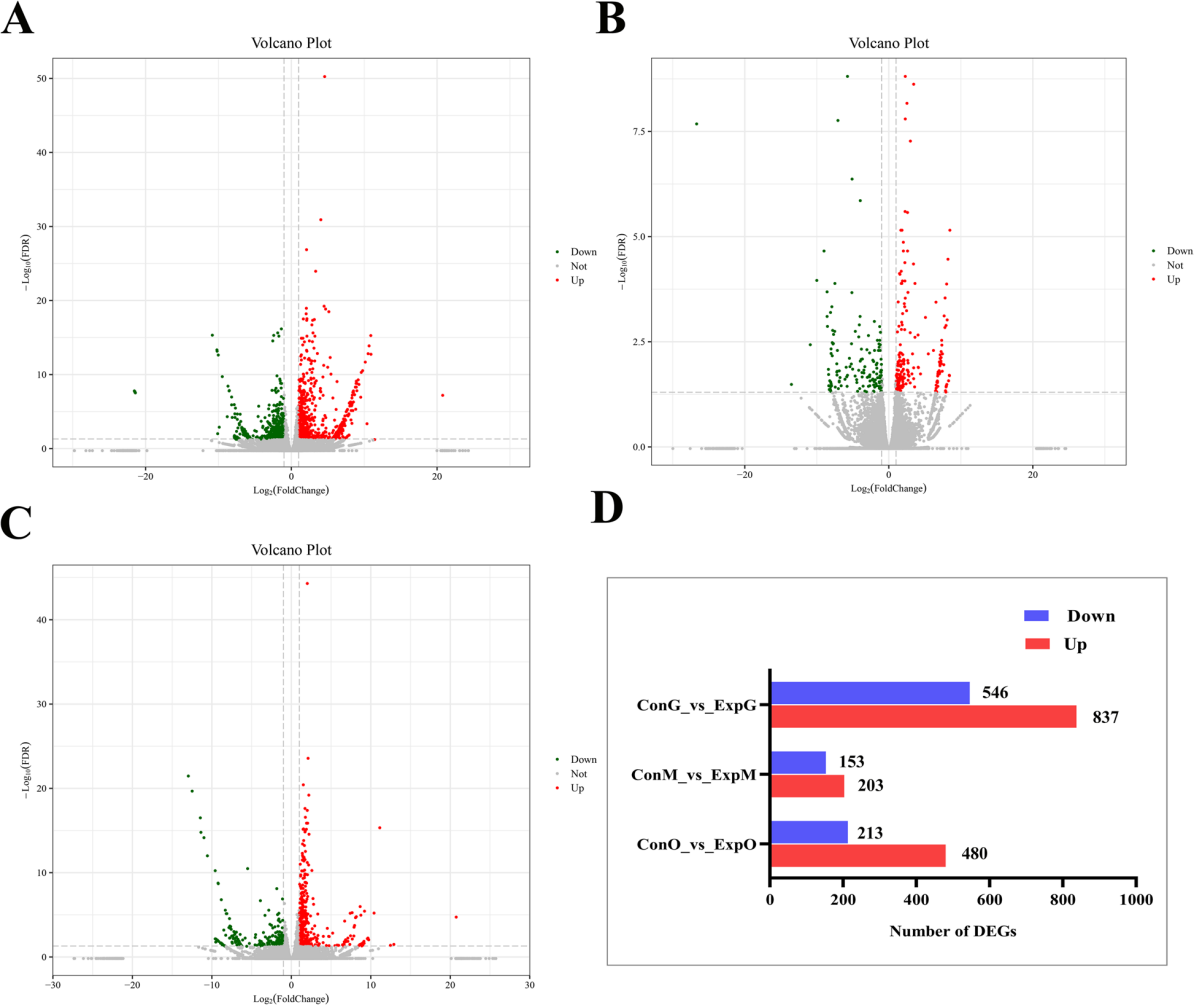
Volcano plots and count charts of the DEGS. Each point in the volcano plots (**A**–**C**) represents a DEG. The x-axis represents the logarithm of the fold difference in expression of a gene in the two experimental groups, the y-axis represents the negative logarithm of the FDR. Green dots represent down-regulated DEGs, red dots represent up-regulated DEGs, and grey dots represent non-DEGs. **A** ConG vs. ExpG; **B** ConM vs. ExpM; **C** ConO vs. ExpO. **D** The number of DEGs in the two experimental groups, with the x-axis indicating the number of DEGs and the y-axis showing the comparison between groups

Differential screening was performed using DESeq2 software, with fold-change ≥ 2 and FDR < 0.05 as the screening criteria for the DEGs. The number of DEGs for ConG vs. ExpG was 1383, including 837 up-regulated and 546 down-regulated genes; the number of DEGs for ConM vs. ExpM was 356, including 203 up-regulated and 153 down-regulated genes; the number of DEGs for ConO vs. ExpO was 693, including 480 up-regulated and 213 down-regulated genes (where ‘Con’ denotes the control group and ‘Exp’ the experimental group, for the different tissues G, M, and O). There were a total of 2532 DEGs in the two experimental groups (Fig. 1).

The DEGs were functionally annotated in seven databases, with a total of 2127 annotated DEGs. 1631, 1495, 936, 527, 1166, 1430, and 1313 DEGs were annotated in the Swiss-Prot, GO, KEGG, COG, KOG, Pfam, and NR databases, respectively (Table 1).

**Table 1:** Differential gene annotation

DEG_Set	Total	Swiss-Prot	GO	KEGG	COG	KOG	Pfam	NR
ConG vs. ExpG	1234	943	873	537	289	681	838	796
ConM vs. ExpM	327	263	236	163	89	189	243	185
ConO vs. ExpO	566	425	386	236	149	296	349	332
Total	2127	1631	1495	936	527	1166	1430	1313

### GO enrichment annotation of the DEGs

**Fig. 2:**
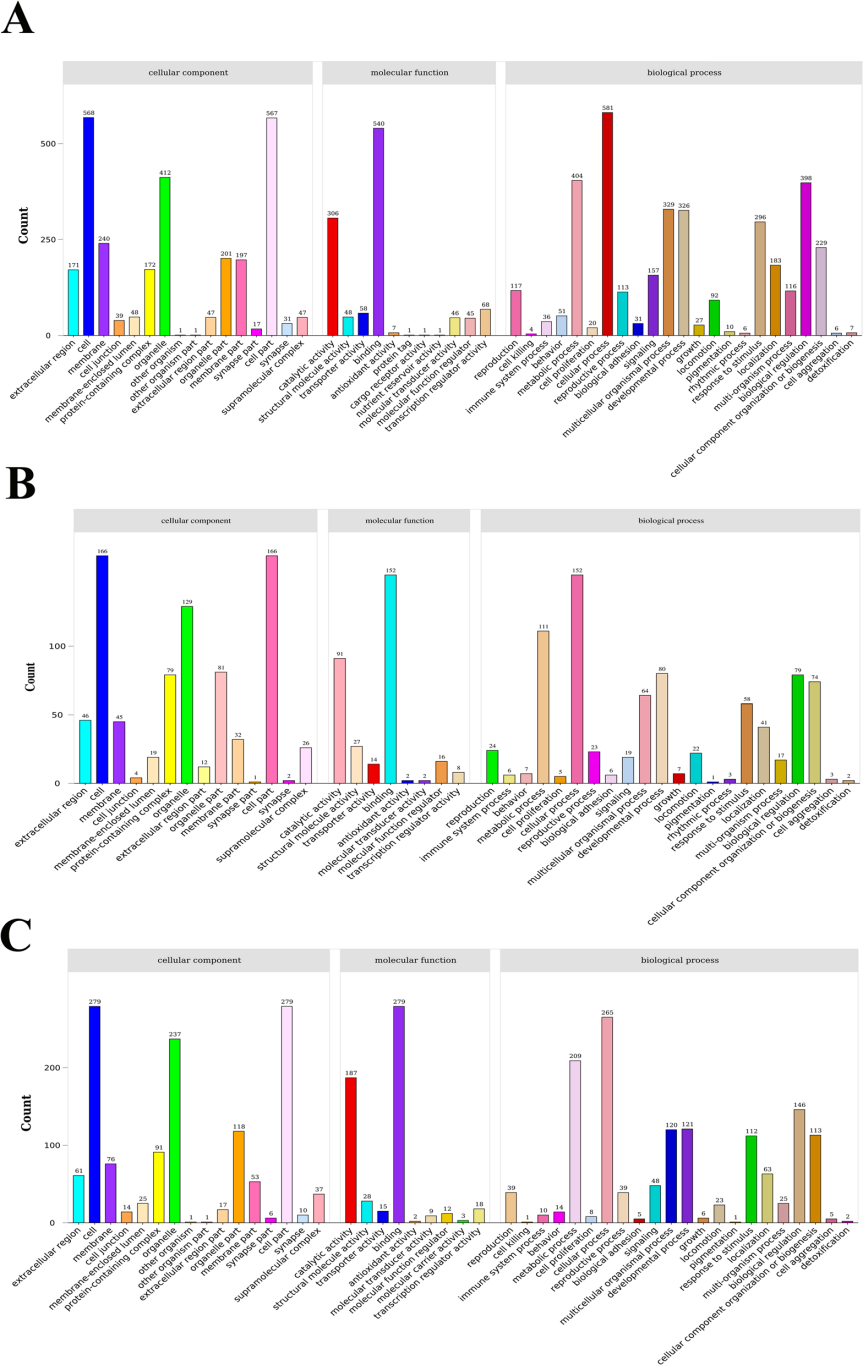
Statistical plot of the GO classification of the DEGs. The horizontal coordinate shows the GO classification and the vertical coordinate shows the number of genes. **A** ConG vs. ExpG; **B** ConM vs. ExpM; **C** ConO vs. ExpO

According to the GO database, DEGs were annotated into three categories: cellular component, molecular functions, and biological processes. There were 873 DEGs in the ConG vs. ExpG group, 236 DEGs in the ConM vs. ExpM group, and 386 DEGs in the ConO vs. ExpO group. Of these, “cell” and “cell part” were representative GO terms in the cellular component classification. “Binding” and “cellular processes” were representative GO terms in the molecular functions and biological processes categories, respectively (Fig. 2).

### KEGG pathway analyses

**Fig. 3:**
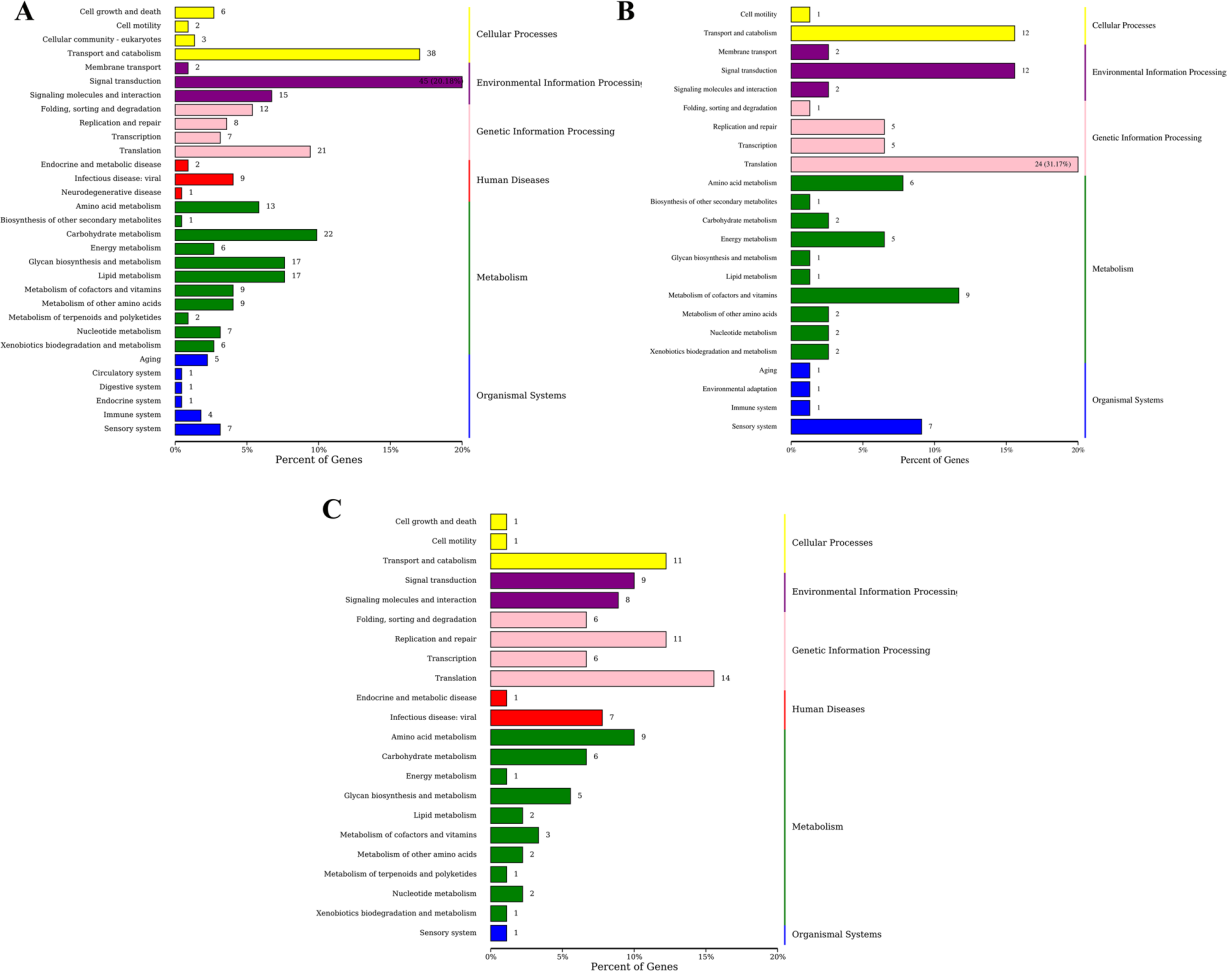
KEGG classification of all DEGs. The left and right y-axes indicate the names of the primary and secondary metabolic pathways, respectively. The x-axis indicates the number and proportion of genes annotated for each pathway. **A** ConG vs. ExpG; **B** ConM vs. ExpM; **C** ConO vs. ExpO

The DEGs in each group were annotated according to the pathway types in the KEGG classification. A total of 537 DEGs were mapped to 112 pathways in ConG vs. ExpG, with the highest percentage (20.18%) appearing in the “Signal transduction” category. A total of 163 DEGs were mapped to 54 different pathways in the ConM vs. ExpM group, with the “Translation” category containing the highest proportion of secondary metabolic pathways. There were 236 DEGs mapped to 66 pathways in the ConO vs. ExpO group, including “Translation” among the representative secondary metabolic pathways (Fig. 3).

### Identification of candidate DEGs regulated by 20E in the different tissues

**Fig. 4:**
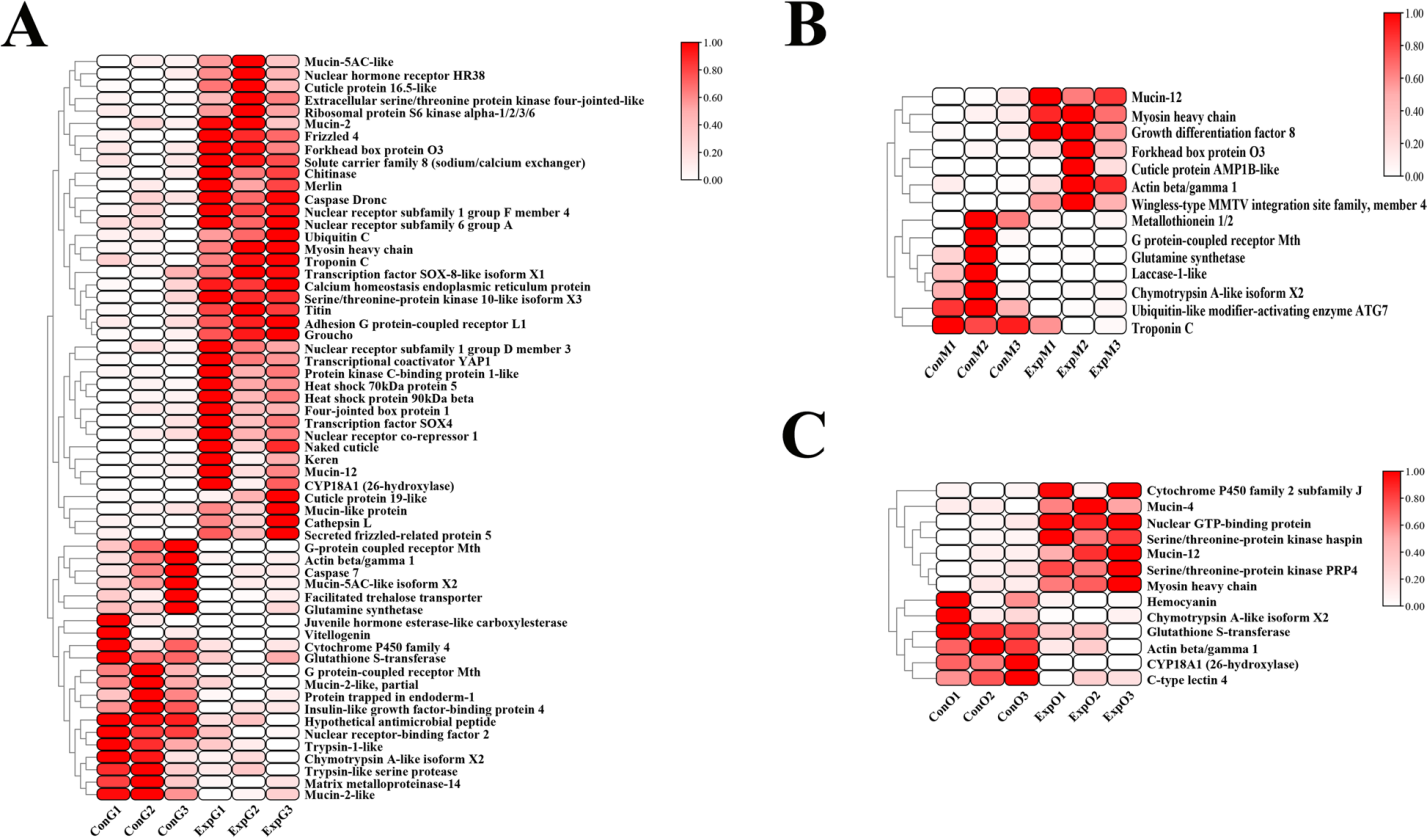
Heatmaps showing the levels of expression of the genes related to molt. The x-axis indicates the grouping of samples, while the y-axes represent the clustering patterns of both the genes and their expression levels. Each row corresponds to a unique gene and the color gradients within the row indicate the relative expression level of that gene. The levels of gene expression are expressed as FPKM (fragments per kilobase million). **A** ConG vs. ExpG; **B** ConM vs. ExpM; **C** ConO vs. ExpO

Numerous DEGs associated with molt or 20E were screened in the transcriptomes of the different tissues in the experimental and control groups. A total of 60 candidate DEGs were identified in the gill transcriptome after injection with 20E, including 39 up-regulated genes and 21 down-regulated genes (Fig. 4A). Fourteen candidate DEGs were screened in the muscle transcriptome, of which seven were up-regulated and seven were down-regulated after 20E injection (Fig. 4B). Compared with the first two groups, a relatively smaller number of candidate genes were screened in the ovary transcriptomes, with 13 candidates identified. (Fig. 4C).

### qRT-PCR validation of the DEGs

**Fig. 5:**
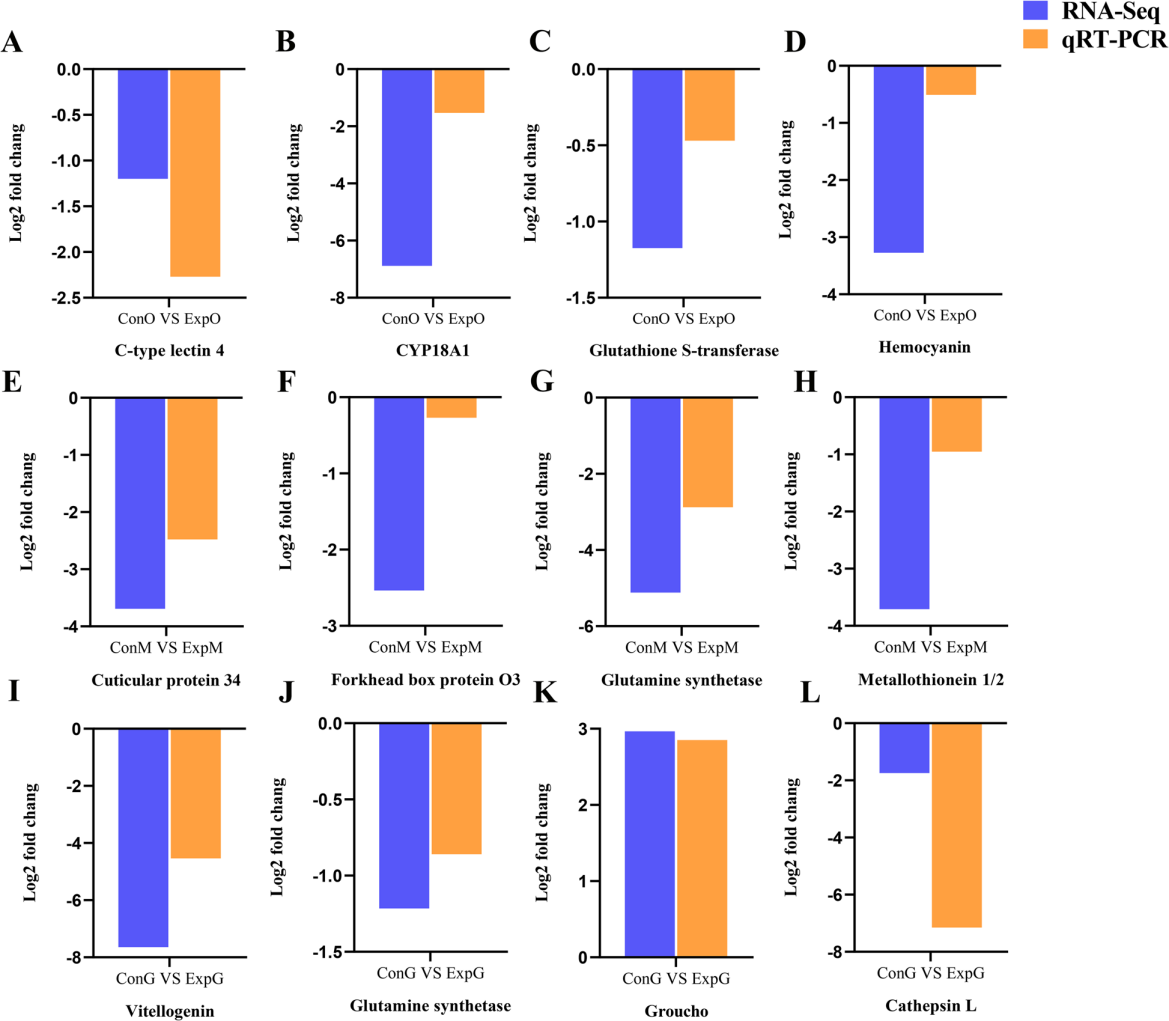
qRT-PCR validation of the RNA sequencing results. **A** C-type lectin 4. **B** CYP18A1. **C** Glutahione S-transferase. **D** Hemocyanin. **E** Cuticular protein 34. **F** Forkhead box protein O3. **G** Glutamine synthetase. **H** Metallothionein 1/2. **I** Vitellogenin. **J** Glutamine synthetase. **K** Groucho. **L** Cathepsin L

To validate the accuracy of the transcriptome data, twelve DEGs were examined and verified by qRT-PCR. The results showed that the qRT-PCR results were consistent with the expression trends of the sequencing results, demonstrating the reliability of the transcriptome data (Fig. 5).

### Expression of important candidate DEGs regulated by 20E at the different stages of molt

**Fig. 6:**
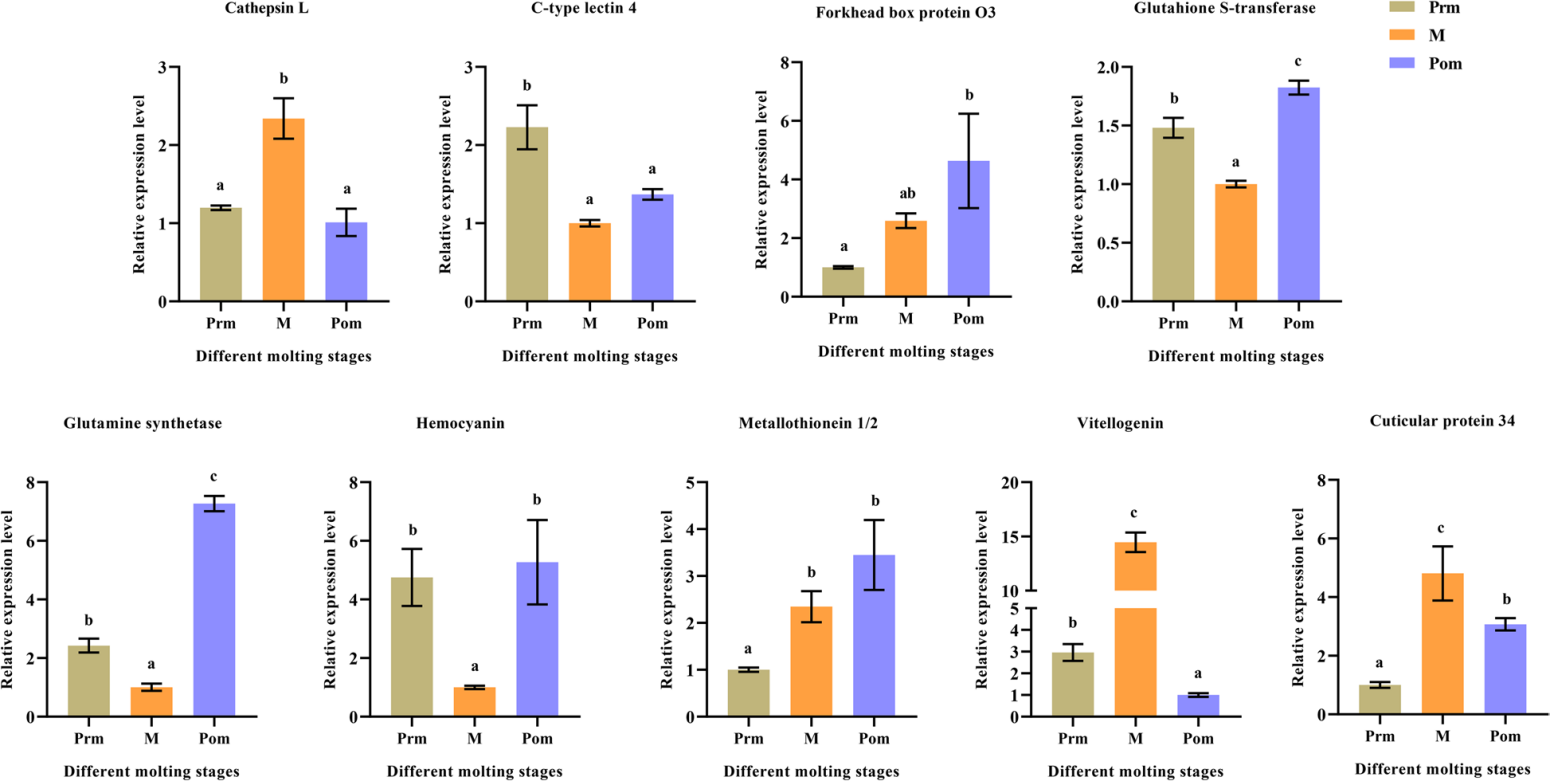
The qRT-PCR results of strong candidate DEGs regulated by 20E during the different stages of molt. The data are presented as the mean ± SD (*n* = 6). Bars with different letters indicate statistically significant differences (*P* < 0.05). Prm, pre-molt; M, mid-molt; Pom, post-molt

A preliminarily investigation of the functions of the 20E-regulated candidate DEGs during molt was performed by further examination of the expression of these genes during the different stages of molt using qRT-PCR (Fig. 6). The results showed that the expression levels of three genes, *Cathepsin L*, *Vg*, and *Cuticular protein 34*, were highest during the mid-molt stage. The expression levels of the *FoxO3* and *Metallothionein 1/2* genes gradually increased from the pre-molt to the post-molt stage. The expression levels of *CTL4, Glutahione S-transferase*, and *Hemocyanin* decreased from the pre-molt stage to mid-molt stage and increased thereafter.

## Discussion

20E plays a critical role in arthropods in regulating molt, promoting growth and development, modulating the reproductive system, and influencing immune responses and is vital for arthropod adaptation, reproduction, and survival. Our previous studies indirectly demonstrated the functional role of 20E in the molting process of *M. nipponense* [10]. Although the significance of 20E in molt has been confirmed, the molecular mechanisms involved remain poorly understood. Therefore, the current study set out to comprehensively understand the mechanistic role of 20E in the molting process of *M. nipponense* using transcriptome experiments involving different tissues. This study is the first to develop a transcriptome library of the different tissues responsive to 20E in *M. nipponense*. A total of 2127 DEGs were identified in the different functional groups, representing genes that exhibited significant differences in expression after 20E treatment, compared with the control group, indicating that 20E is involved in a variety of biological responses.

In the present study, after exogenous injection of 20E, the experimental and control groups had the highest number of DEGs in the gill tissue, followed by the ovary, with muscle having the fewest. The gills of decapod crustaceans play a primary role in maintaining their ion balance through osmoregulation [24, 25]. Various studies have shown that the Y-organ is located on the ventral and anterior sides of the gill chamber in crustaceans [26]. Our previous studies have demonstrated that the *Spook* gene, which catalyzes 20E synthesis, is highly expressed in the gill tissue of *M. nipponense* [10]. We therefore speculate that the gill tissue is closely related to the Y-organ and plays a significant role in the regulation of 20E. Studies of insects have shown that in the larval stages, the precursor ecdysteroids of 20E are synthesized in the prothoracic gland, whereas in adults they are mainly synthesized in the ovaries [27]. It has been shown that in some insects there is a positive correlation between 20E and reproduction [28, 29]. In this study, a large number of DEGs were also identified in the ovarian transcriptome of *M. nipponense* after exogenous injection of 20E. This also provides preliminary evidence of a close association between the ovary and 20E. Molt is the process whereby arthropods grow, and involves shedding of the old exoskeleton and the formation of a new one. Muscles play a crucial role in crustacean molt. The muscles perform a series of rhythmic, repetitive contractions, involving the swallowing of air and the intake of water [30]. However, our transcriptome results show that muscles have the fewest genes that respond to 20E. We speculate that the transmission of the 20E signal is primarily performed in the other tissues and then manifests in the muscles.

The DEGs in the gill tissue were primarily enriched in the Signal transduction pathway. Signal transduction is an important process in cellular communication and involves a series of signaling molecules, protein kinases, intracellular signaling pathways, and transcription factors, among others. We found significant enrichments of genes in the Signal transduction related pathways, possibly because they encode for critical proteins and regulatory factors associated with the signal transduction processes. The Translation pathway was significantly enriched with DEGs in the ovarian and muscle tissues. Translation is the process by which proteins are synthesized by converting nucleotide sequences in RNA molecules into proteins with specific amino acid sequences. The DEGs were enriched in the translation related pathways, maybe because they encode for regulatory factors and functional proteins involved in protein synthesis and play significant roles in the rate and quality of protein synthesis, among other things. The following discussion revolves around the important candidate genes and their relevance in the context of the diverse biological responses associated with 20E.

*Matrix metalloproteinase 2* is critical in ovulation and corpus luteum formation in *Drosophila*, and ecdysteroid signaling is essential for its activation during these processes [6, 31]. Our transcriptome results showed that *Metalloproteinase 1/2* was up-regulated after 20E injection, demonstrating that 20E also has an activating effect on it in *M. nipponense*. In *Helicoverpa armigera*, 20E promotes proteolysis during molt by activating *Forkhead box O* (*FoxO*) [32]. Similarly, 20E induced *FoxO* upregulation to promote lipolysis during *Bombyx* spp. molt and pupation [33]. In the current study, *Forkhead box protein O3* (*FoxO3*) was also induced by 20E, so we speculate that *FoxO3* may perform a similar function during molt in *M. nipponense*. Previous studies have shown that Cathepsins are essential during insect development and metamorphosis and participate in the digestion and degradation of tissues [34]. 20E induces *cathepsin L* expression involved in the destruction of fat bodies [35]. Similar to previous reports, 20E injection in *M. nipponense* significantly up-regulated the expression of *cathepsin L*. Knockdown of *cathepsin L* resulted in significantly stunted growth or delayed molt in *Caenorhabditis elegans* larvae [36]. We speculate that *cathepsin L* may also exercise an important function during crustacean molt. Following a blood meal, mosquitoes trigger the secretion of ecdysone in their ovaries. This ecdysone undergoes hydroxylation, transforming into 20E, which subsequently activates the transcription of the *Vitellogenin* (*Vg*) gene [37]. The current results show that 20E significantly upregulates *Vg* genes in *M. nipponense*. Previous studies have shown that 20E plays a major role in vitellogenesis during female insect reproduction [29]. In *Nilaparvata lugens*, the inhibition of the *Groucho* gene impedes the transformation of nymphs into adults and leads to mortality during the molting process [38]. Furthermore, administering ecdysone to fifth instar nymphs resulted in a notable decrease in *Groucho* expression, corroborating our findings [38]. Because *Groucho* exhibits a similar response to 20E regulation in *M. nipponense*, we speculate that it may also be involved in the molting process. Cuticle proteins are an important component of the exoskeleton in arthropods. Previous reports indicate that a number of cuticle protein genes are regulated by 20E [39]. In *Tenebrio molitor*, exogenous treatment with 20E resulted in a decrease in cuticular protein 20, while cuticular protein 22 expression increased by 2.5 times [40]. This indicates that the various cuticle proteins are differentially regulated by 20E [41]. C-type lectins are crucial in insect innate immunity. In the fat body, 20E serves as a broad inhibitor of innate immunity [42]. In *H. armigera*, 20E caused down-regulation of the expression of C-type lectin 4 (*CTL4*) [43], whereas in the current study 20E significantly induced the expression of this gene. Therefore, we speculate that there may be species differences in the regulatory effect of 20E on *CTL4*. *CYP18A1* plays a crucial role in *Drosophila melanogaster* metamorphosis by deactivating 20E [44]. Administration of 20E in *M. nipponense* significantly induced the expression of *CYP18A1*, indicating that 20E has a positive regulatory effect on this gene. In *Trichinella spiralis*, glutamine synthetase is involved in larval molt and development [45]. Hemocyanin facilitates the synthesis of new exoskeletons in crustaceans and insects [46]. In general, these candidate genes are regulated by 20E and may play important roles in molt and other physiological activities in *M. nipponense*.

In summary, the current study established the first transcriptome library of the various tissues of *M. nipponense* in response to 20E. In the main, this study revealed the dominant role of 20E in the molting process of *M. nipponense*. The potential significance of this study to the cultivation of *M. nipponense* lies in highlighting the possibility of utilizing 20E to regulate the molting process, providing a potential means of improving the efficiency of shrimp cultivation and shrimp growth. In addition, the study identified a large number of candidate DEGs regulated by 20E, further confirming its widespread regulatory role in molt. These genes may be closely associated with the regulatory network related to molt, and are vital for a deeper understanding of the molecular regulatory mechanisms of 20E. Therefore, these results hold potential practical value for improving the aquacultural management of *M. nipponense* and can provide guidance by which farmers can optimize molt cycle management, stimulate production, and enhance aquacultural efficiency.

### Supplementary Information

**Additional file 1.**

## Data Availability

The datasets generated during the current study are available in the [NCBI] repository, [http://www.ncbi.nlm.nih.gov/bioproject/1014997].
